# A Review of Fractional Order Entropies

**DOI:** 10.3390/e22121374

**Published:** 2020-12-05

**Authors:** António M. Lopes, José A. Tenreiro Machado

**Affiliations:** 1 LAETA/INEGI, Faculty of Engineering, University of Porto, Rua Dr. Roberto Frias, 4200-465 Porto, Portugal; 2 Department of Electrical Engineering, Institute of Engineering, Polytechnic of Porto, Rua Dr. António Bernardino de Almeida, 431, 4249-015 Porto, Portugal; jtm@isep.ipp.pt

**Keywords:** fractional calculus, entropy, information theory

## Abstract

Fractional calculus (FC) is the area of calculus that generalizes the operations of differentiation and integration. FC operators are non-local and capture the history of dynamical effects present in many natural and artificial phenomena. Entropy is a measure of uncertainty, diversity and randomness often adopted for characterizing complex dynamical systems. Stemming from the synergies between the two areas, this paper reviews the concept of entropy in the framework of FC. Several new entropy definitions have been proposed in recent decades, expanding the scope of applicability of this seminal tool. However, FC is not yet well disseminated in the community of entropy. Therefore, new definitions based on FC can generalize both concepts in the theoretical and applied points of view. The time to come will prove to what extend the new formulations will be useful.

## 1. Introduction

In recent decades, the generalization of the concepts of differentiation [1,2,3,4] and entropy [5,6,7,8] have received considerable attention. In the first case we may cite the fractional calculus (FC) [9,10]. FC was introduced by Leibniz in the scope of mathematics by the end of the 17th century, but only recently found application in biology [11,12], physics [13,14] and engineering [15,16], among others [17,18]. The concept of entropy was introduced by Clausius [19] and Boltzmann [20] in the field of thermodynamics. Later, entropy was also explored by Shannon [21] and Jaynes [22] in the context of information theory. Meanwhile, both topics evolved considerably, motivating the formulation of fractional operators [23,24] and entropy indices [25,26,27,28,29,30,31,32,33,34,35,36,37,38]. These generalizations extend the application of the two mathematical tools and highlight certain characteristics, such as the power-law behavior, non-locality and long range memory [39,40].

This paper reviews the concept of entropy in the framework of FC. In fact, FC is not yet well disseminated among the community of entropy and, therefore, new definitions based on FC may expand the scope of this powerful tool. To the authors’ best knowledge, new entropy definitions are welcomed by the scientific community, somehow contrary to what happens with recent fractional operators. Consequently, the manuscript does not intend to assess the pros or the cons of the distinct formulations for some given problem. In a similar line of thought, the analysis of entropy-based indices proposed in the literature for comparing or characterizing some phenomena or probability distributions are outside the focus of this paper. Interested readers can obtain further information on divergence measures [41] and mutual information [42], as well as for sample [43], approximate [44], permutation [45], spectral [46], and fuzzy [47] entropies, among others [48]. Indeed, the main idea of this paper is to review the concept of fractional entropy and to present present day state of its development.

The paper is organized as follows. Section 2 presents the fundamental concepts of FC. Section 3 introduces different entropies with one, two and three parameters. Section 4 reviews the fractional-order entropy formulations. Section 5 compares the different formulations for four well-known distributions. Section 6 assesses the impact of the fractional entropies and analyses their main areas of application. Finally, Section 7 outlines the main conclusions.

## 2. Fractional-Order Derivatives and Integrals

FC models capture non-local effects, useful in the study of phenomena with long range correlations in time or space.

Let us consider the finite interval [a,b], with a,b∈R and a<b, and let n−1<q<n, with n∈N. The Euler’s gamma function is denoted by Γ(·) and the operator [·] calculates the integer part of the argument. Several definitions of fractional derivatives were formulated [24,49,50]. A small set is presented in the follow-up, which includes both historically relevant and widely used definitions:

- The left-side and the right-side Caputo derivatives,
  (1) $$\begin{array}{c} {}^{C}D_{a^{+}}^{q}f\left(t\right)=\frac{1}{\Gamma (n-q)}\int_{a}^{x}\frac{1}{{\left(x-\tau \right)}^{q-n+1}}\frac{d^{n}}{d\tau^{n}}f\left(\tau \right)d\tau ,\;\;x\geq a, \end{array}$$
  (2) $$\begin{array}{c} {}^{C}D_{b^{-}}^{q}f\left(x\right)=\frac{{\left(-1\right)}^{n}}{\Gamma (n-q)}\int_{x}^{b}\frac{1}{{\left(\tau -x\right)}^{q-n+1}}\frac{d^{n}}{d\tau^{n}}f\left(\tau \right)d\tau ,\;\;x\leq b, \end{array}$$
- The left-side and the right-side Grünwald-Letnikov derivatives,
  (3) $$\begin{array}{c} {}^{GL}D_{a^{+}}^{q}f\left(x\right)=\lim_{h\to 0}\;h^{-q}\sum_{m=0}^{\left[\frac{x-a}{h}\right]}{\left(-1\right)}^{m}\left(\begin{array}{c} q\\ m \end{array}\right)f\left(x-mh\right),\;\;x\geq a, \end{array}$$
  (4) $$\begin{array}{c} {}^{GL}D_{b^{-}}^{q}f\left(x\right)=\lim_{h\to 0}\;h^{-q}\sum_{m=0}^{\left[\frac{b-x}{h}\right]}{\left(-1\right)}^{m}\left(\begin{array}{c} q\\ m \end{array}\right)f\left(x+mh\right),\;\;x\leq b, \end{array}$$
- The Hadamard derivative,
  (5) $$\begin{array}{c} {}^{Ha}D_{+}^{q}f\left(x\right)=\frac{q}{\Gamma (1-q)}\int_{0}^{x}\frac{f(x)-f(\tau )}{{\left[\log\left(x/\tau \right)\right]}^{q+1}}\frac{d\tau }{\tau }, \end{array}$$
- The left-side and right-side Hilfer derivatives of type 0≤β≤1,
  (6) $$\begin{array}{c} {}^{H}D_{a^{+}}^{q}f\left(x\right){=}^{RL}I_{a^{+}}^{\gamma -q}{\frac{d^{n}}{dx^{n}}}^{RL}I_{a^{+}}^{\left(1-\beta )(n-q\right)}f\left(x\right), \end{array}$$
  (7) $$\begin{array}{c} {}^{H}D_{b^{-}}^{q}f\left(x\right){=}^{RL}I_{b^{-}}^{\gamma -q}\left(-1\right){\frac{d^{n}}{dx^{n}}}^{RL}I_{b^{-}}^{\left(1-\beta )(n-q\right)}f\left(x\right), \end{array}$$
  where ${}^{RL}I_{a^{+}}^{q}$ and ${}^{RL}I_{b^{-}}^{q}$ denote the left-side and right-side Riemann-Liouville fractional integrals of order q>0, respectively, defined by:
  (8) $$\begin{array}{c} {}^{RL}I_{a^{+}}^{q}f\left(x\right)=\frac{1}{\Gamma (q)}\int_{a}^{x}\frac{f(\tau )}{{\left(x-\tau \right)}^{1-q}}d\tau ,\;\;x\geq a, \end{array}$$
  (9) $$\begin{array}{c} {}^{RL}I_{b^{-}}^{q}f\left(x\right)=\frac{1}{\Gamma (q)}\int_{x}^{b}\frac{f(\tau )}{{\left(\tau -x\right)}^{1-q}}d\tau ,\;\;x\leq b, \end{array}$$
- The Karcı derivative
  (10) $${}^{K}D^{q}f\left(x\right)=\lim_{h\to 0}\frac{\frac{d\{{\left[f\left(x+h\right)\right]}^{q}-{\left[f\left(x\right)\right]}^{q}\}}{dh}}{\frac{d[{\left(x+h\right)}^{q}-x^{q}]}{dh}}=\frac{\frac{d}{dx}\left[f\left(x\right)\right]\;\cdot \;\left[f{\left(x\right)}^{q-1}\right]}{x^{q-1}}.$$
- The Liouville, the left-side and the right-side Liouville derivatives,
  (11) $$\begin{array}{c} {}^{L}D^{q}f\left(x\right)=\frac{1}{\Gamma (1-q)}\frac{d}{dx}\int_{-\infty }^{x}\frac{f(\tau )}{{\left(x-\tau \right)}^{q}}d\tau ,\;\;-\infty <x<+\infty , \end{array}$$
  (12) $$\begin{array}{c} {}^{L}D_{0^{+}}^{q}f\left(x\right)=\frac{1}{\Gamma (n-q)}\frac{d^{n}}{dx^{n}}\int_{0}^{x}\frac{f(\tau )}{{\left(x-\tau \right)}^{q-n+1}}d\tau ,\;\;x>0, \end{array}$$
  (13) $$\begin{array}{c} {}^{L}D_{0^{-}}^{q}f\left(x\right)=\frac{{\left(-1\right)}^{n}}{\Gamma (n-q)}\frac{d^{n}}{dx^{n}}\int_{x}^{+\infty }\frac{f(\tau )}{{\left(x-\tau \right)}^{q-n+1}}d\tau ,\;\;x<+\infty , \end{array}$$
- The Marchaud, the left-side and the right-side Marchaud derivatives,
  (14) $$\begin{array}{c} {}^{M}D_{}^{q}f\left(x\right)=\frac{q}{\Gamma (1-q)}\int_{-\infty }^{x}\frac{f(x)-f(\tau )}{{\left(x-\tau \right)}^{q+1}}d\tau , \end{array}$$
  (15) $$\begin{array}{c} {}^{M}D_{+}^{q}f\left(x\right)=\frac{q}{\Gamma (1-q)}\int_{0}^{+\infty }\frac{f(x)-f(x-\tau )}{\tau^{q+1}}d\tau , \end{array}$$
  (16) $$\begin{array}{c} {}^{M}D_{-}^{q}f\left(x\right)=\frac{q}{\Gamma (1-q)}\int_{0}^{+\infty }\frac{f(x)-f(x+\tau )}{\tau^{q+1}}d\tau , \end{array}$$
- The left-side and the right-side Riemann-Liouville derivatives,
  (17) $$\begin{array}{c} {}^{RL}D_{a^{+}}^{q}f\left(x\right)=\frac{1}{\Gamma (n-q)}\frac{d^{n}}{dx^{n}}\int_{a}^{x}\frac{f(\tau )}{{\left(x-\tau \right)}^{q-n+1}}d\tau ,\;\;x\geq a, \end{array}$$
  (18) $$\begin{array}{c} {}^{RL}D_{b^{-}}^{q}f\left(x\right)=\frac{{\left(-1\right)}^{n}}{\Gamma (n-q)}\frac{d^{n}}{dx^{n}}\int_{x}^{b}\frac{f(\tau )}{{\left(\tau -x\right)}^{q-n+1}}d\tau ,\;\;x\leq b, \end{array}$$
- The Riesz derivative,
  (19) $$\begin{array}{c} {}^{R}D_{x}^{q}f\left(x\right)=-\frac{1}{2\cos(q\pi /2)}\frac{1}{\Gamma (q)}\frac{d^{n}}{dx^{n}}\left\{\int_{-\infty }^{x}\frac{f(\tau )}{{\left(x-\tau \right)}^{q-n+1}}d\tau +\int_{x}^{+\infty }\frac{f(\tau )}{{\left(\tau -x\right)}^{q-n+1}}d\tau \right\}, \end{array}$$
- The local Yang derivative,
  (20) $$\begin{array}{c} {}^{Y}D_{-}^{q}f{\left(x\right)}_{|x=x_{0}}=\lim_{x\to x_{0}}\frac{\Delta^{q}[f\left(x\right)-f\left(x_{0}\right]}{{\left(x-x_{0}\right)}^{q}}. \end{array}$$

Often, the Caputo formulation is applied in physics and numerical integration, the Riemann-Liouville in calculus, and the Grünwald-Letnikov in engineering, signal processing and control. These classical definitions are the most frequently used by researchers. In what concerns the mathematical pros and cons of the Karcı and the Yang derivatives, readers may visit [23,51] and references therein. In fact, it should be noted that some formulations need some careful reflection and are the matter of some controversy, since many authors do not consider them as fractional operators [23,52,53]. Nevertheless, the debate about what it really means the term ‘fractional derivative’ is still ongoing among contemporary mathematicians [51].

## 3. The Concept of Entropy

Let us consider a discrete probability distribution $P=\{p_{1},p_{2},\cdots ,p_{N}\}$, with $\sum_{i}p_{i}=1$ and $p_{i}\geq 0$. The Shannon entropy, $S^{\left(S\right)}$, of distribution P is defined as:

(21) $$S^{\left(S\right)}=\sum_{i}p_{i}I\left(p_{i}\right)=-\sum_{i}p_{i}\ln p_{i},$$

and represents the expected value of the information content given by $I\left(p_{i}\right)=-\ln p_{i}$. Therefore, for the uniform probability distribution we have $p_{i}=N^{-1}$, N∈N, and the Shannon entropy takes its maximum value S=lnN, yielding the Boltzmann formula, up to a multiplicative factor, *k*, which denotes the Boltzmann constant.

The Rényi and Tsallis entropies are one-parameter generalizations of (21) given by, respectively:

(22) $$S_{q}^{\left(R\right)}=\frac{1}{1-q}\ln\left(\sum_{i}p_{i}^{q}\right),\;q>0,q\neq 1,$$

(23) $$S_{q}^{\left(T\right)}=\frac{1}{q-1}\left(1-\sum_{i}p_{i}^{q}\right),\;q\in R.$$

The entropies $S_{q}^{\left(R\right)}$ and $S_{q}^{\left(T\right)}$ reduce to the Shannon formulation $S^{\left(S\right)}$ when q→1. The Rényi entropy has an inverse power law equilibrium distribution [54], satisfying the zero-th law of thermodynamics [55]. It is important in statistics and ecology to quantify diversity, in quantum information to measure entanglement, and in computer science for randomness extraction. The Tsallis entropy was proposed in the scope of nonextensive statistical mechanics and has found application in the field of complex dynamics, in diffusion equations [56] and Fokker-Planck systems [57].

Other one-parameter entropies are the Landsberg-Vedral and Abe formulations [26,58]:

(24) $$S_{q}^{\left(L\right)}=\frac{1}{1-q}\left(\frac{1}{\sum_{i}p_{i}^{q}}-1\right),$$

(25) $$S_{q}^{\left(A\right)}=-\sum_{i}\frac{p_{i}^{q}-p_{i}^{q^{-1}}}{q-q^{-1}},\;q\in ]0,1].$$

Expression (24) is related to the Tsallis entropy by $S_{q}^{\left(L\right)}=\frac{S_{q}^{\left(T\right)}}{\sum_{i}p_{i}^{q}}$, and is often known as normalized Tsallis entropy. Expression (25) is a symmetric modification of the Tsallis entropy, which is invariant to the exchange $q↔q^{-1}$, and we have $S_{q}^{\left(A\right)}=\frac{\left(q-1\right)S_{q}^{\left(T\right)}-q^{-1}S_{q^{-1}}^{\left(T\right)}}{q-q^{-1}}$.

The two-parameter Sharma-Mittal entropy [32] is a generalization of the Shannon, Tsallis and Rényi entropies, and is defined as follows:

(26) $$S_{r,q}^{\left(SM\right)}=\frac{1}{1-r}\left[{\left(\sum_{i}p_{i}^{q}\right)}^{\frac{1-r}{1-q}}-1\right],\;q>0,q\neq 1,r\neq 1.$$

The Sharma-Mittal entropy reduces to the Rényi, Tsallis and Shannon’s formulations for the limits r→1, r→q and {r,q}→{1,1}, respectively.

Examples of three-parameter formulations consist of the gamma and the Kaniadakis entropies, $S_{d,c_{1},c_{2}}^{\left(G\right)}$ and $S_{\kappa ,\tau ,\zeta }^{\left(K\right)}$, respectively. The gamma entropy is given by [35]:

(27) $$S_{d,c_{1},c_{2}}^{\left(G\right)}=\sum_{i}\frac{e}{c_{2}-c_{1}}\Gamma \left(d+1,1-c_{1}\ln p_{i},1-c_{2}\ln p_{i}\right),$$

where *e* denotes the Napier constant, $\Gamma (a,z_{1},z_{2})$ represents the generalized incomplete gamma function, defined by:

(28) $$\Gamma \left(a,z_{1},z_{2}\right)=\Gamma \left(a,z_{1}\right)-\Gamma \left(a,z_{2}\right)=\int_{z_{1}}^{z_{2}}t^{a-1}e^{-t}dt$$

and $\Gamma \left(x,y\right)=\int_{y}^{\infty }t^{x-1}e^{-t}dt$ is the upper incomplete gamma function.

The entropy $S_{d,c_{1},c_{2}}^{\left(G\right)}$ follows the first three Khinchin axioms [35,59,60] within the parameter regions defined by (29) and (30):

(29) $$c_{2}>1>c_{1}>0,\;1-\frac{1}{c_{1}}<d<1-\frac{1}{c_{2}},$$

(30) $$c_{1}>1>c_{2}>0,\;1-\frac{1}{c_{2}}<d<1-\frac{1}{c_{1}}.$$

Different combinations of the parameters yield distinct entropy formulations [35]. For example, if we set $\left\{d,c_{1},c_{2}\right\}=\left\{0,1,q\right\}$, then we recover the Tsallis entropy, while for $\left\{d,c_{1},c_{2}\right\}=\left\{0,1\pm \epsilon ,1\mp \epsilon \right\}$, ϵ→0, we obtain the Shannon entropy.

The Kaniadakis entropy belongs to a class of trace-form entropies given by [38]:

(31) $$S=-\sum_{i}p_{i}\Lambda \left(p_{i}\right),$$

where Λ(x) is a strictly increasing function defined for positive values of the argument, noting that $\Lambda (x\to 0^{+})=-\infty$. The function Λ(x) can be viewed as a generalization of the ordinary logarithm [38] that, for three-parameter, yields:

(32) $$\Lambda \left(x\right)=\ln_{\kappa ,\tau ,\zeta }\left(x\right)=\frac{\zeta^{\kappa }x^{\tau +\kappa }-\zeta^{-\kappa }x^{\tau -\kappa }-\zeta^{\kappa }+\zeta^{-\kappa }}{\left(\kappa +\tau \right)\zeta^{\kappa }+\left(\kappa -\tau \right)\zeta^{-\kappa }}.$$

Therefore, the Kaniadakis entropy, $S_{\kappa ,\tau ,\zeta }^{\left(K\right)}$, can be expressed as:

(33) $$S_{\kappa ,\tau ,\zeta }^{\left(K\right)}=-\sum_{i}p_{i}\ln_{\kappa ,\tau ,\zeta }\left(p_{i}\right),\;\kappa ,\zeta \in R,|\kappa |-1<\tau \leq |\kappa |.$$

The Entropy $S_{\kappa ,\tau ,\zeta }^{\left(K\right)}$ is Lesche [61] and thermodynamically [62] stable for −|κ|≤τ≤|κ|. Distinct combinations of the parameters yield several entropy formulations [38]. For example, if we set κ=τ=(q−1)/2 or κ→0, τ=0, then expression (33) yields the Tsallis and the Shannon entropies, respectively.

Other entropies can be found in the literature [63,64], but a thorough review of all proposed formulations is out of the scope of this paper.

## 4. Fractional Generalizations of Entropy

It was noted [65] that the Shannon and Tsallis entropies have the same generating function $\sum_{i}p_{i}^{x}$ and that the difference in the Formulas (21) and (23) is just due to the adopted differentiation operator. In fact, using the standard first-order differentiation, $\frac{d}{dx}$, we obtain the Shannon entropy:

(34) $$S^{\left(S\right)}=\lim_{x\to -1}\frac{d}{dx}\sum_{i}p_{i}^{-x},$$

while adopting the Jackson *q*-derivative [66], $D_{q}f\left(x\right)=\frac{f(qx)-f(x)}{qx-x}$, 0<q<1, yields the Tsallis entropy [28]:

(35) $$S_{q}^{\left(T\right)}=\lim_{x\to -1}D_{q}\sum_{i}p_{i}^{-x}.$$

Other expressions for entropy can be obtained by adopting additional differentiation operators.

In 2001, Akimoto and Suzuki [67] proposed the one-parameter fractional entropy, $S_{\alpha }^{\left(AS\right)}$, given by:

(36) $$S_{\alpha }^{\left(AS\right)}=-\lim_{x\to 1}\sum_{i}\frac{d^{\alpha }}{dx^{\alpha }}e^{x\ln p_{i}},$$

where $\frac{d^{\alpha }}{dx^{\alpha }}{=}^{RL}D_{a^{+}}^{\alpha }$ is the Riemann-Liouville operator (17), with a=0.

The expressions (36) and (17) yield:

(37) $$S_{\alpha }^{\left(AS\right)}=\sum_{i}\frac{\alpha -1}{\Gamma (2-\alpha )}{}_{1}F_{1}\left(1;1-\alpha ;\ln p_{i}\right),\;0<\alpha <1,$$

where ${}_{1}F_{1}\left(a;b;c\right)$ denotes the confluent hypergeometric function of the first kind [68]:

(38) $${}_{1}F_{1}\left(a;b;x\right)=1+\frac{a}{b}\frac{x}{1!}+\frac{a(a+1)}{b(b+1)}\frac{x^{2}}{2!}+\frac{a(a+1)(a+2)}{b(b+1)(b+2)}\frac{x^{3}}{3!}+\cdots .$$

It can be shown that [67] has the concavity and non-extensivity properties. In the limit α→1, it obeys positivity and gives the Shannon entropy, $S^{\left(S\right)}$.

In 2009, Ubriaco introduced a one-parameter fractional entropy, $S_{\alpha }^{\left(U\right)}$, given by [69]:

(39) $$S_{\alpha }^{\left(U\right)}=\lim_{x\to -1}\frac{d}{dx}\left({}^{RL}D_{-\infty }^{\alpha -1}\sum_{i}e^{-x\ln p_{i}}\right),$$

where ${}^{RL}D_{-\infty }^{\alpha -1}$ is the Riemann-Liouville left-side derivative (17) with a→−∞.

Therefore, we obtain:

(40) $$S_{\alpha }^{\left(U\right)}=\lim_{x\to -1}\frac{d}{dx}\frac{1}{\Gamma (1-\alpha )}\sum_{i}\int_{-\infty }^{x}\frac{e^{-\tau \ln p_{i}}}{{\left(x-\tau \right)}^{\alpha }}d\tau .$$

Performing the integration and taking the limit x→−1, it yields:

(41) $$S_{\alpha }^{\left(U\right)}=\sum_{i}p_{i}{\left(-\ln p_{i}\right)}^{\alpha },\;0\leq \alpha \leq 1.$$

The Ubriaco entropy (41) is thermodynamically stable and obeys the same properties of the Shannon entropy, with the exception of additivity. When α→1, we recover the Shannon entropy.

In 2012, Yu et al. [70] formulated a one-parameter fractional entropy by means of the simple expression:

(42) $$S_{\alpha }^{\left(Y\right)}={-}^{RL}I_{0}^{\alpha }\left(p_{i}\ln p_{i}\right),\;\alpha \in R^{+},$$

where the operator ${}^{RL}I_{0}^{\alpha }$ is the left-side Riemann-Liouville integral (8), with a=0. Expression (42) obeys the concavity property and is an extension and generalization of the Shannon entropy.

Another fractional entropy was derived in 2014 by Radhakrishnan et al. [71], being given by:

(43) $$S_{q,\alpha }^{\left(RCJ\right)}=\sum_{i}p_{i}^{q}{\left(-\ln p_{i}\right)}^{\alpha },\;q,\alpha >0.$$

The two-parameter expression (43) was inspired in (41) and the entropy (44) derived by Wang in the context of the incomplete information theory [72]:

(44) $$S_{q}^{\left(W\right)}={\langle -\ln p_{1}\rangle }_{q}=\sum_{i}p_{i}^{q}\left(-\ln p_{i}\right),$$

where $\sum_{i}p_{i}^{q}=1$ and ${\langle O\rangle }_{q}=\sum_{i}p_{i}^{q}O_{i}$ denotes the *q*-expectation that characterizes incomplete normalization.

The entropy (43) is considered a fractional entropy in a fractal phase space in which the parameters *q* and α are associated with fractality and fractionality, respectively. In the limit, when (i) q→1 Equation (43) reduces to (41), (ii) α→1 recovers $S_{q}^{\left(W\right)}$, and (iii) {q,α}={1,1} expression (43) yields the standard Shannon formula (21).

In 2014, Machado followed a different line of thought [73], thinking of Shannon information $I\left(p_{i}\right)=-\ln p_{i}$ as a function of order zero lying between the integer-order cases $D^{-1}I\left(p_{i}\right)=p_{i}\left(1-\ln p_{i}\right)$ and $D^{1}I\left(p_{i}\right)=-\frac{1}{p_{i}}$. In the perspective of FC, this observation motivated the formulation of information and entropy of order α∈R as [24]:

(45) $$I_{\alpha }\left(p_{i}\right)=D^{\alpha }I\left(p_{i}\right)=-\frac{p_{i}^{-\alpha }}{\Gamma \left(\alpha +1\right)}\left(\ln p_{i}+\tilde{\psi }\right),$$

(46) $$S_{\alpha }^{\left(M\right)}=\sum_{i}\left[-\frac{p_{i}^{-\alpha }}{\Gamma \left(\alpha +1\right)}\left(\ln p_{i}+\tilde{\psi }\right)\right]p_{i},$$

where $D^{\alpha }$ denotes a fractional derivative operator, $\tilde{\psi }=\psi \left(1\right)-\psi \left(1-\alpha \right)$ and $\psi \left(\;\cdot \;\right)$ represent the digamma function.

The one-parameter fractional entropy (46) fails to obey some of the Khinchin axioms with exception of the case q=0 that leads to the Shannon entropy [74]. This behavior is in line with what occurs in FC, where fractional derivatives fail to obey some of the properties of integer-order operators [1].

Expression (46) was generalized by Jalab et al. [75] in the framework of local FC [76]. A adopting (20), the following expression was proposed:

(47) $$S_{\alpha }^{\left(J\right)}=\sum_{i}\left[-\frac{p_{i}^{-i\alpha }}{\Gamma \left(i\alpha +1\right)}\left(\ln p_{i}+\tilde{\psi }\right)\right]p_{i}.$$

Equation (47) decreases from 1 to 1−α, α∈]0,1[. Therefore, we have:

(48) $$S_{\alpha }^{\left(J\right)}\approx \sum_{i}\left[-\frac{p_{i}^{-i\alpha }}{\Gamma \left(i\alpha +1\right)}\left(\ln p_{i}+\frac{1}{\alpha }\right)\right]p_{i}.$$

In 2016, Karcı [77] proposed the fractional derivative (10), based on the concept of indefinite limit and the l’Hôpital’s rule. Adopting $f\left(x\right)=\sum_{i}p_{i}^{-x}$, and using (10) into (34), he derived the following expression for fractional entropy [78]:

(49) $$S_{\alpha }^{\left(K\right)}=\left|{}^{K}D^{\alpha }f\left(x\right)\right|=\left|{}^{K}D^{\alpha }\sum_{i}p_{i}^{-x}\right|=\sum_{i}p_{i}\left|{\left(\frac{p^{-x}}{x}\right)}^{\alpha -1}\left(-1\right)p^{-1}\ln p\right|=\sum_{i}p_{i}\left|{\left(-p\right)}^{\alpha }\ln p\right|.$$

In 2019, Ferreira and Machado [79] presented a new formula for the entropy based on the work of Abe [65] and Ubriaco [69]. They start by the definition of left-side Liouville fractional derivative of a function *f* with respect to another function *g*, with $g^{\prime }>0$, given by:

(50) $${}^{L}D_{g}^{\alpha }f\left(x\right)=\frac{1}{\Gamma \left(1-\alpha \right)g^{\prime }\left(x\right)}\frac{d}{dx}\int_{-\infty }^{x}{\left[g\left(x\right)-g\left(s\right)\right]}^{-\alpha }g^{\prime }\left(s\right)f\left(s\right)ds,\;0<\alpha \leq 1.$$

Choosing $f\left(x\right)=p_{i}^{-x}$ and $g\left(x\right)=e^{x+1}$ expression (50) leads to:

(51) $$\begin{array}{cc} {}^{L}D_{g}^{\alpha }f\left(x\right) & =\frac{1}{\Gamma \left(1-\alpha \right)e^{x+1}}\frac{d}{dx}\int_{-\infty }^{x}{\left[e^{x+1}-e^{s+1}\right]}^{-\alpha }e^{s+1}e^{-s\ln(p_{i})}ds \end{array}$$

(52) $$\begin{array}{cc} \; & =\left[1-\alpha -\ln\left(p_{i}\right)\right]e^{\left(-\alpha -1\right)\left(x+1\right)+x[1-\ln\left(p_{i}\right)]+1}\frac{\Gamma (1-\ln\left(p_{i}\right))}{\Gamma (2-\alpha -\ln\left(p_{i}\right))}. \end{array}$$

Therefore, we have:

(53) $${}^{L}D_{g}^{\alpha }f\left(-1\right)=\left[1-\alpha -\ln\left(p_{i}\right)\right]p_{i}\frac{\Gamma (1-\ln\left(p_{i}\right))}{\Gamma (2-\alpha -\ln\left(p_{i}\right))},$$

which applying Γ(x+1)=xΓ(x), for x>0, results in:

(54) $${}^{L}D_{g}^{\alpha }f\left(-1\right)=p_{i}\frac{\Gamma (1-\ln\left(p_{i}\right))}{\Gamma (1-\alpha -\ln\left(p_{i}\right))}.$$

Using (54) into (34) gives:

(55) $$S_{\alpha }^{\left(FM\right)}=\sum_{i}p_{i}\frac{\Gamma (1-\ln\left(p_{i}\right))}{\Gamma (1-\alpha -\ln\left(p_{i}\right))},\;0<\alpha \leq 1.$$

In 2019, Machado and Lopes [80] proposed two fractional formulations of the Rényi entropy, $S_{q,\alpha }^{\left(ML_{1}\right)}$ and $S_{q,\alpha }^{\left(ML_{2}\right)}$. Their derivation adopts a general averaging operator, instead of the linear one that is assumed for the Shannon entropy (21). Let us consider a monotonic function f(x) with inverse $f^{-1}\left(x\right)$. Therefore, for a set of real values $\left\{x_{i}\right\}$, i=1,2,⋯, with probabilities $\left\{p_{i}\right\}$, we can define a general mean [81] associated with f(x) as:

(56) $$f^{-1}\left(\sum_{i}p_{i}f\left(x_{i}\right)\right).$$

Applying (56) to the Shannon entropy (21) we obtain:

(57) $$S=f^{-1}\left(\sum_{i}p_{i}f\left(I\left(p_{i}\right)\right)\right),$$

where f(x) is a Kolmogorov–Nagumo invertible function [82]. If the postulate of additivity for independent events is considered in (56), then only two functions f(x) are possible, consisting of $f_{1}\left(x\right)=c\;\cdot \;x$ and $f_{2}\left(x\right)=c\;\cdot \;\exp\left[(1-q)x\right]$, with c,q∈R. For $f_{1}\left(x\right)$ we get the ordinary mean and we verify that $S=S^{\left(S\right)}$. For $f\left(x\right)=c\;\cdot \;e^{(1-q)x}$ we have the expression:

(58) $$S=\frac{1}{1-q}\sum_{i}p_{i}\;\cdot \;\exp\left[\left(1-q\right)I\left(p_{i}\right)\right],$$

which gives the Rényi entropy:

(59) $$S_{q}^{\left(R\right)}=\frac{1}{1-q}\ln\left(\sum_{i}p_{i}^{q}\right),\;q>0,q\neq 1.$$

If we combine (45) and (58), then we obtain:

(60) $$\begin{array}{cc} S_{q,\alpha }^{\left(ML_{1}\right)} & =\frac{1}{1-q}\ln\left\{\sum_{i}p_{i}\;\cdot \;\exp\left[\left(1-q\right)\;\cdot \;I_{\alpha }\left(p_{i}\right)\right]\right\}\\ \; & =\frac{1}{1-q}\ln\left\{\sum_{i}p_{i}\;\cdot \;\exp\left[\left(q-1\right)\;\cdot \;\frac{p_{i}^{-\alpha }}{\Gamma \left(\alpha +1\right)}\left(\ln p_{i}+\tilde{\psi }\right)\right]\right\}. \end{array}$$

On the other hand, if we rewrite (22) as:

(61) $$\begin{array}{cc} S_{q}^{\left(R\right)} & =\frac{q}{1-q}\ln\left[{\left(\frac{1}{N}\sum_{i}p_{i}^{q}\right)}^{\frac{1}{q}}\;\cdot \;N^{\frac{1}{q}}\right]\\ \; & =\frac{q}{1-q}\ln\left[{\langle p_{i}\rangle }_{g}\;\cdot \;N^{\frac{1}{q}}\right], \end{array}$$

where ${\langle p_{i}\rangle }_{g}={\left(\frac{1}{N}\sum_{i}p_{i}^{q}\right)}^{\frac{1}{q}}$ is a generalized mean, then we obtain:

(62) $$S_{q,\alpha }^{\left(ML_{2}\right)}=D^{\alpha }H_{q}^{\left(R\right)}=\frac{1}{N^{\frac{\alpha }{q}}}\frac{q}{1-q}\left[\frac{{\langle p_{i}\rangle }_{g}^{-\alpha }}{\Gamma \left(\alpha +1\right)}\left(\frac{1}{q}\ln N+\ln{\langle p_{i}\rangle }_{g}+\tilde{\psi }\right)\right].$$

In the limit, when α→0, both $S_{q,\alpha }^{\left(ML_{1}\right)}$ and $S_{q,\alpha }^{\left(ML_{2}\right)}$ yield (22).

## 5. Comparison of the Fractional-Order Entropies

In this section we use the fractional entropy formulas to compute the entropy both of abstract and real-world data series.

### 5.1. Fractional-Order Entropy of Some Probability Distributions

We calculate the entropy of four well-known probability distributions, namely those of Poisson, Gaussian, Lévy and Weibull. We consider these cases just with the purpose of illustrating the behavior of the different formulations. Obviously other cases could be considered, but we limit the number for the sake of parsimony. Firstly, we present the results obtained with the one-parameter entropies $S_{\alpha }^{\left(AS\right)}$, $S_{\alpha }^{\left(U\right)}$, $S_{\alpha }^{\left(Y\right)}$, $S_{\alpha }^{\left(M\right)}$, $S_{\alpha }^{\left(J\right)}$, $S_{\alpha }^{\left(K\right)}$ and $S_{\alpha }^{\left(FM\right)}$. Then, we consider the two-parameter formulations $S_{q,\alpha }^{\left(RCJ\right)}$, $S_{q,\alpha }^{\left(ML_{1}\right)}$ and $S_{q,\alpha }^{\left(ML_{2}\right)}$. Table 1 summarizes the constants adopted for the distributions and the intervals of variation of the entropy parameters.

**Figure 1:**
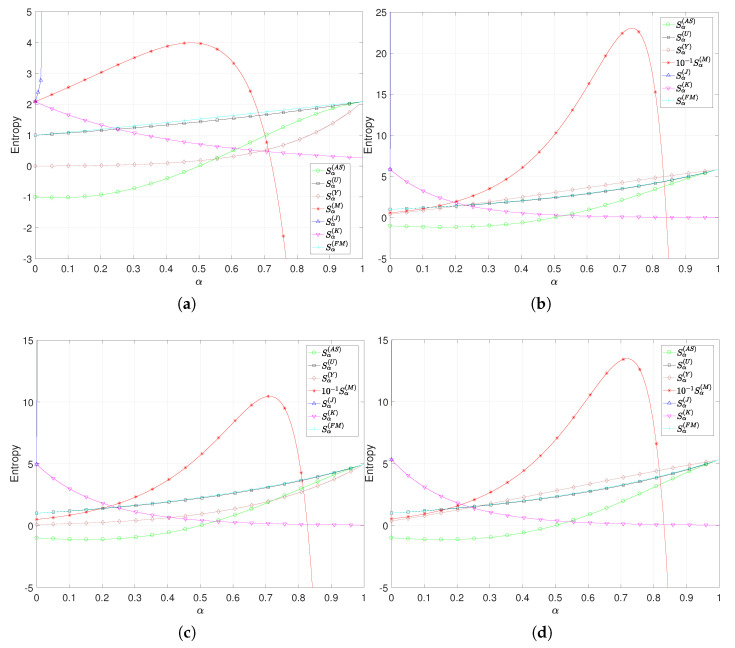
The values of $S_{\alpha }^{\left(AS\right)}$, $S_{\alpha }^{\left(U\right)}$, $S_{\alpha }^{\left(Y\right)}$, $S_{\alpha }^{\left(M\right)}$, $S_{\alpha }^{\left(J\right)}$, $S_{\alpha }^{\left(K\right)}$ and $S_{\alpha }^{\left(FM\right)}$ versus α∈[0,1] for the (**a**) Poisson, (**b**) Gaussian, (**c**) Lévy and (**d**) Weibull distributions.

Figure 1 depicts the values of $S_{\alpha }^{\left(AS\right)}$, $S_{\alpha }^{\left(U\right)}$, $S_{\alpha }^{\left(Y\right)}$, $S_{\alpha }^{\left(M\right)}$, $S_{\alpha }^{\left(J\right)}$, $S_{\alpha }^{\left(K\right)}$ and $S_{\alpha }^{\left(FM\right)}$ versus α∈[0,1]. We verify that in the limits, either α→0 or α→1, the values of the Shannon entropy are calculated as 2.087, 5.866, 4.953 and 5.309, respectively. Moreover, it can be seen that $S_{\alpha }^{\left(U\right)}$ and $S_{\alpha }^{\left(FM\right)}$ are very close to each other, $S_{\alpha }^{\left(AS\right)}$ does not obey positivity, $S_{\alpha }^{\left(J\right)}$ diverges at small values of α, $S_{\alpha }^{\left(M\right)}$ has a maximum at values of α close to 0.6 and diverges as α→1.

**Figure 2:**
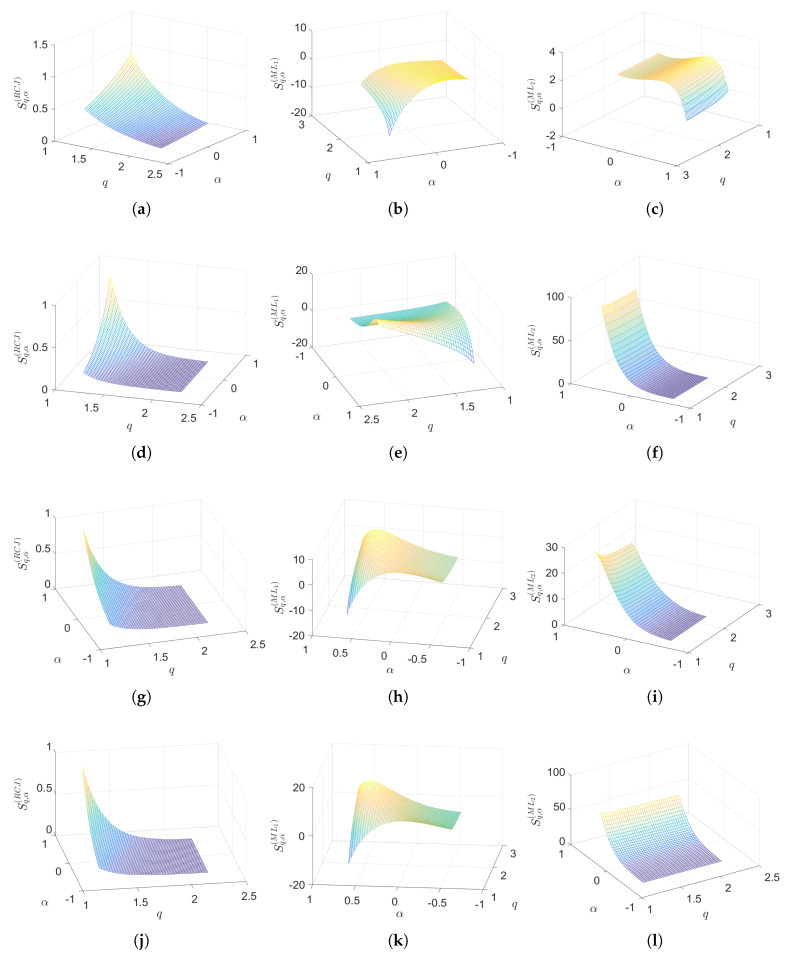
The values of $S_{q,\alpha }^{\left(RCJ\right)}$, $S_{q,\alpha }^{\left(ML_{1}\right)}$ and $S_{q,\alpha }^{\left(ML_{2}\right)}$ versus α∈[−0.6,0.6] and q∈[1.2,2.2] for the (**a**–**c**) Poisson, (**d**–**f**) Gaussian, (**g**–**i**) Lévy and (**j**–**l**) Weibull distributions.

Figure 2 portraits the values of $S_{q,\alpha }^{\left(RCJ\right)}$, $S_{q,\alpha }^{\left(ML_{1}\right)}$ and $S_{q,\alpha }^{\left(ML_{2}\right)}$ versus α∈[−0.6,0.6] and q∈[1.2,2.2]. We verify that in the domain considered the entropies vary slightly and do not diverge.

### 5.2. Fractional-Order Entropy of Real-World Data

We calculate the entropy of a real-world time series, namely the Dow Jones Industrial Average (DJIA) financial index. The DJIA raw data are available at the Yahoo Finance website (https://finance.yahoo.com/). Herein, we consider the stock closing values in the time period from 1 January 1987 up to 24 November 2018, with one-day sampling interval. Occasional missing values, as well as values corresponding to closing days, are estimated using linear interpolation. The processed DJIA time series, $x=\{x_{1},x_{2},\cdots ,x_{T}\}$, T=12,381 points, is used to construct a histogram of relative frequencies, f(x), with N=50 bins equally spaced and non-overlapping, for estimating the probability distribution of *x*.

**Figure 3:**
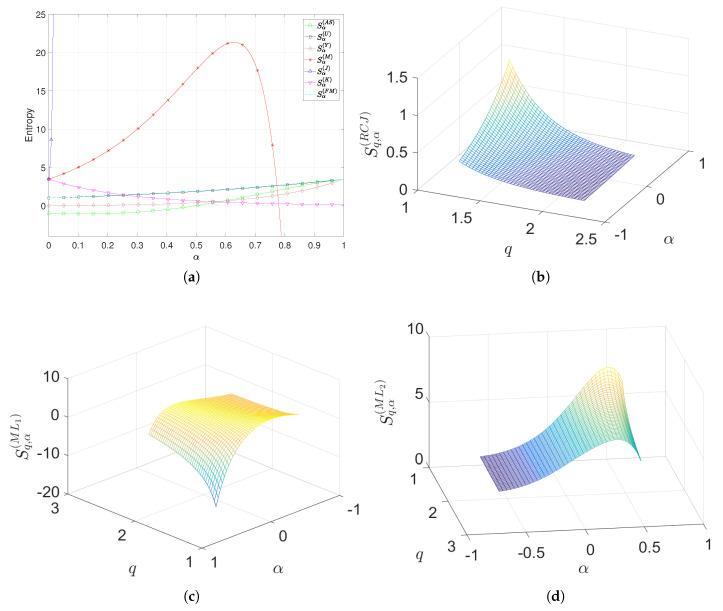
The entropy of the DJIA stock index for daily closing values in the time period from 1 January 1987 up to 24 November 2018, with one-day sampling interval: (**a**) $S_{\alpha }^{\left(AS\right)}$, $S_{\alpha }^{\left(U\right)}$, $S_{\alpha }^{\left(Y\right)}$, $S_{\alpha }^{\left(M\right)}$, $S_{\alpha }^{\left(J\right)}$, $S_{\alpha }^{\left(K\right)}$ and $S_{\alpha }^{\left(FM\right)}$ versus α∈[0,1]; (**b**–**d**) $S_{q,\alpha }^{\left(RCJ\right)}$, $S_{q,\alpha }^{\left(ML_{1}\right)}$ and $S_{q,\alpha }^{\left(ML_{2}\right)}$ versus α∈[−0.6,0.6] and q∈[1.2,2.2].

Figure 3a depicts the values of $S_{\alpha }^{\left(AS\right)}$, $S_{\alpha }^{\left(U\right)}$, $S_{\alpha }^{\left(Y\right)}$, $S_{\alpha }^{\left(M\right)}$, $S_{\alpha }^{\left(J\right)}$, $S_{\alpha }^{\left(K\right)}$ and $S_{\alpha }^{\left(FM\right)}$ versus α∈[0,1]. We verify that, as shown in Section 5.1, $S_{\alpha }^{\left(U\right)}$ and $S_{\alpha }^{\left(FM\right)}$ yield similar results, $S_{\alpha }^{\left(J\right)}$ diverges for small values of α, and $S_{\alpha }^{\left(M\right)}$ has a maximum at values of α close to 0.6, diverging when α→1.

Figure 3b–d show the values of $S_{q,\alpha }^{\left(RCJ\right)}$, $S_{q,\alpha }^{\left(ML_{1}\right)}$ and $S_{q,\alpha }^{\left(ML_{2}\right)}$ versus α∈[−0.6,0.6] and q∈[1.2,2.2], yielding results of the same type as before.

## 6. Impact and Applications of the Fractional-Order Entropies

To assess the impact of the fractional-order entropies on the scientific community, we consider the number of citations received by the nine papers that first proposed them. Table 1 summarizes the results obtained from the database Scopus on 7 November 2020 (www.scopus.com). We verify that those nine papers were cited 218 times by 170 distinct papers, and that the expressions proposed by Ubriaco and Machado received more attention.

**Figure 4:**
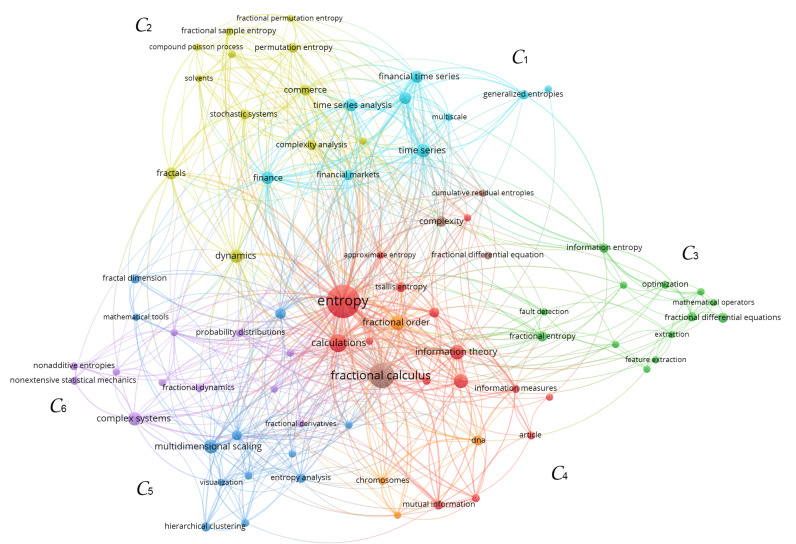
The map of co-occurrence of the authors’ keywords in the 170 papers extracted from Scopus for constructing Table 2. The minimum value of co-occurrence of each keyword is 3. The clusters are represented by $C=\{C_{1},\cdots ,C_{6}\}$.

To unravel the main areas of application of the fractional entropies, we use the VOSviewer (https://www.vosviewer.com/), which allows the construction and visualization of bibliometric networks [83]. The bibliometric data of the 170 papers that cite the nine papers that present the fractional-order entropies were collected from Scopus for constructing Table 2, and are the input information to the VOSviewer. The co-occurrence of the authors’ keywords in the 170 papers is analyzed, with the minimum value of co-occurrence of each keyword set to 3. Figure 4 depicts the generated map. We verify the emergence of six clusters, $C=\{C_{1},\ldots ,C_{6}\}$. At the top, the light-blue cluster, $C_{1}$, includes the fields of finance and finance time series analysis, while the light-green one, $C_{2}$, encompasses a variety of areas, such as solvents, fractals, commerce, and stochastic systems, tightly connected to some entropy-based complexity measures. On the right, the dark-green cluster, $C_{3}$, includes the areas of fault detection and image processing. At the bottom of the map, the red cluster, $C_{4}$, highlights the fields of chromosome and DNA analysis, while the dark-blue, $C_{5}$, one emphasizes some clustering and visualization techniques, as multidimensional scaling and hierarchical clustering. On the left, the magenta cluster, $C_{6}$, includes keywords not related with applications.

In summary, we conclude that the fractional entropies were applied to a considerable number of distinct scientific areas and that we may foresee a promising future for their development by exploring the synergies of the two mathematical tools. The prevalence of some proposals, from the point of view of citations, may be due to the time elapsed since their formulation. Indeed, more recent formulations had not yet sufficient time to disseminate in the community. Another reason may have to do with the type and audience of journal where they were published. Nonetheless, a full bibliometric analysis is not the leitmotif of the present paper.

## 7. Conclusions

This paper reviewed the concept of entropy in the framework of FC. To the best of the authors’ knowledge the fractional entropies proposed so far were included in this review. The different formulations result from the adopted (i) fractional-order operator or (ii) generating function. In general such entropies are non-extensive and converge to the classical Shannon entropy for certain values of their parameters. The fractional entropies have found applications in the area of complex systems, where the classical formulations revealed some limitations. The FC brings a shinny future in further developments of entropy and its applications.

## Figures and Tables

**Table 1:** The constants adopted for the probability distributions and the intervals of variation of the entropy parameters.

Distribution	Expression	Parameters	Domain	Order 1-par. Entropy	Order 2-par. Entropy
Poisson	$f\left(x\right)\frac{\lambda^{z}e^{-\lambda }}{z!}$	λ=4	z=0,1,…,50	α∈[0,1]	α∈[−0.6,0.6]
Gaussian	$f\left(x\right)=\frac{1}{\sigma \sqrt{2\pi }}e^{-\frac{1}{2}{\left(\frac{x-\mu }{\sigma }\right)}^{2}}$	σ=4 μ=0	x∈[−2,2]		
Lévy	$f\left(x\right)=\sqrt{\frac{c}{2\pi }}\frac{e^{-\frac{c}{\left(2x-\mu \right)}}}{{\left(x-\mu \right)}^{\left(3/2\right)}}$	c=4 μ=0	x∈[0.1,20]		q∈[1.2,2.2]
Weibull	$f\left(x\right)=\frac{k}{\lambda }{\left(\frac{x}{\lambda }\right)}^{\left(k-1\right)}e^{-{\left(x/\lambda \right)}^{k}}$	k=1.5 λ=1	x∈[0.01,2.5]		

**Table 2:** Citations received by the nine papers that proposed the fractional-order entropies, according to the database Scopus on 7 November 2020.

Entropy	Equation Number	Authors	Reference	N. Citations	Year
$S_{\alpha }^{\left(AS\right)}$	(37)	Akimoto and Suzuki	[67]	5	2001
$S_{\alpha }^{\left(U\right)}$	(41)	Ubriaco	[69]	88	2009
$S_{\alpha }^{\left(Y\right)}$	(42)	Yu et al.	[70]	7	2012
$S_{q,\alpha }^{\left(RCJ\right)}$	(43)	Radhakrishnan et al.	[71]	3	2014
$S_{\alpha }^{\left(M\right)}$	(46)	Machado	[73]	79	2014
$S_{\alpha }^{\left(J\right)}$	(47)	Jalab et al.	[75]	6	2019
$S_{\alpha }^{\left(K\right)}$	(49)	Karcı	[77]	16	2016
$S_{\alpha }^{\left(FM\right)}$	(55)	Ferreira and Machado	[79]	4	2019
$S_{q,\alpha }^{\left(ML_{1}\right)}$	(60)	Machado and Lopes	[80]	5	2019
$S_{q,\alpha }^{\left(ML_{2}\right)}$	(62)	Machado and Lopes	[80]	5	2019

## References

[1] Oldham K., Spanier J. (1974). The Fractional Calculus: Theory and Application of Differentiation and Integration to Arbitrary Order.

[2] Samko S., Kilbas A., Marichev O. (1993). Fractional Integrals and Derivatives: Theory and Applications.

[3] Miller K., Ross B. (1993). An Introduction to the Fractional Calculus and Fractional Differential Equations.

[4] Kilbas A., Srivastava H., Trujillo J. (2006). Theory and Applications of Fractional Differential Equations.

[5] Plastino A., Plastino A.R. (1999). Tsallis entropy and Jaynes’ Information Theory formalism. Braz. J. Phys..

[6] Li X., Essex C., Davison M., Hoffmann K.H., Schulzky C. (2003). Fractional Diffusion, Irreversibility and Entropy. J. Non-Equilib. Thermodyn..

[7] Mathai A., Haubold H. (2007). Pathway model, superstatistics, Tsallis statistics, and a generalized measure of entropy. Phys. A Stat. Mech. Appl..

[8] Anastasiadis A. (2012). Special Issue: Tsallis Entropy. Entropy.

[9] Tenreiro Machado J.A., Kiryakova V., Kochubei A., Luchko Y. (2019). Recent history of the fractional calculus: Data and statistics. Handbook of Fractional Calculus with Applications: Basic Theory.

[10] Machado J.T., Galhano A.M., Trujillo J.J. (2014). On development of fractional calculus during the last fifty years. Scientometrics.

[11] Ionescu C. (2013). The Human Respiratory System: An Analysis of the Interplay between Anatomy, Structure, Breathing and Fractal Dynamics.

[12] Lopes A.M., Machado J.T. (2014). Fractional order models of leaves. J. Vib. Control..

[13] Hilfer R. (2000). Application of Fractional Calculus in Physics.

[14] Tarasov V. (2010). Fractional Dynamics: Applications of Fractional Calculus to Dynamics of Particles, Fields and Media.

[15] Parsa B., Dabiri A., Machado J.A.T., Baleanu D., Lopes A.M. (2019). Application of Variable order Fractional Calculus in Solid Mechanics. Handbook of Fractional Calculus with Applications: Applications in Engineering, Life and Social Sciences, Part A.

[16] Lopes A.M., Machado J.A.T., Baleanu D., Lopes A.M. (2019). Fractional-order modeling of electro-impedance spectroscopy information. Handbook of Fractional Calculus with Applications: Applications in Engineering, Life and Social Sciences, Part A.

[17] Valério D., Ortigueira M., Machado J.T., Lopes A.M., Petráš I. (2019). Continuous-time fractional linear systems: Steady-state behaviour. Handbook of Fractional Calculus with Applications: Applications in Engineering, Life and Social Sciences, Part A.

[18] Tarasov V.E. (2019). On history of mathematical economics: Application of fractional calculus. Mathematics.

[19] Clausius R., Van Voorst J. (1867). The Mechanical Theory of Heat: With Its Applications to the Steam-Engine and to the Physical Properties of Bodies.

[20] Boltzmann L., Barth J.A. (1897). Vorlesungen über die Principe der Mechanik.

[21] Shannon C.E. (1948). A mathematical theory of communication. Bell Syst. Tech. J..

[22] Jaynes E.T. (1957). Information theory and statistical mechanics. Phys. Rev..

[23] Ortigueira M.D., Machado J.T. (2015). What is a fractional derivative?. J. Comput. Phys..

[24] Valério D., Trujillo J.J., Rivero M., Machado J.T., Baleanu D. (2013). Fractional calculus: A survey of useful formulas. Eur. Phys. J. Spec. Top..

[25] Lopes A.M., Tenreiro Machado J., Galhano A.M. (2015). Multidimensional Scaling Visualization Using Parametric Entropy. Int. J. Bifurc. Chaos.

[26] Landsberg P.T., Vedral V. (1998). Distributions and channel capacities in generalized statistical mechanics. Phys. Lett. A.

[27] Beck C. (2009). Generalised information and entropy measures in physics. Contemp. Phys..

[28] Tsallis C. (1988). Possible generalization of Boltzmann-Gibbs statistics. J. Stat. Phys..

[29] Kaniadakis G. (2002). Statistical mechanics in the context of special relativity. Phys. Rev. E.

[30] Naudts J. (2004). Generalized thermostatistics based on deformed exponential and logarithmic functions. Phys. A Stat. Mech. Appl..

[31] Abe S., Beck C., Cohen E.G. (2007). Superstatistics, thermodynamics, and fluctuations. Phys. Rev. E.

[32] Sharma B.D., Mittal D.P. (1975). New nonadditive measures of entropy for discrete probability distributions. J. Math. Sci..

[33] Wada T., Suyari H. (2007). A two-parameter generalization of Shannon–Khinchin axioms and the uniqueness theorem. Phys. Lett. A.

[34] Bhatia P. (2010). On certainty and generalized information measures. Int. J. Contemp. Math. Sci..

[35] Asgarani S. (2013). A set of new three-parameter entropies in terms of a generalized incomplete Gamma function. Phys. A Stat. Mech. Appl..

[36] Hanel R., Thurner S. (2011). A comprehensive classification of complex statistical systems and an axiomatic derivation of their entropy and distribution functions. EPL (Europhys. Lett.).

[37] Sharma B.D., Taneja I.J. (1975). Entropy of type (*α*, *β*) and other generalized measures in information theory. Metrika.

[38] Kaniadakis G. (2009). Maximum entropy principle and power-law tailed distributions. Eur. Phys. J. B-Condens. Matter Complex Syst..

[39] Tarasov V.E. (2013). Lattice model with power-law spatial dispersion for fractional elasticity. Cent. Eur. J. Phys..

[40] Nigmatullin R., Baleanu D. (2013). New relationships connecting a class of fractal objects and fractional integrals in space. Fract. Calc. Appl. Anal..

[41] Lin J. (1991). Divergence measures based on the Shannon entropy. IEEE Trans. Inf. Theory.

[42] Cover T.M., Thomas J.A. (1991). Entropy, relative entropy and mutual information. Elem. Inf. Theory.

[43] Ebrahimi N., Pflughoeft K., Soofi E.S. (1994). Two measures of sample entropy. Stat. Probab. Lett..

[44] Pincus S.M. (1991). Approximate entropy as a measure of system complexity. Proc. Natl. Acad. Sci. USA.

[45] Bandt C., Pompe B. (2002). Permutation entropy: A natural complexity measure for time series. Phys. Rev. Lett..

[46] Pan Y., Chen J., Li X. (2009). Spectral entropy: A complementary index for rolling element bearing performance degradation assessment. Proc. Inst. Mech. Eng. Part C J. Mech. Eng. Sci..

[47] Fan J.L., Ma Y.L. (2002). Some new fuzzy entropy formulas. Fuzzy Sets Syst..

[48] Rosso O.A., Blanco S., Yordanova J., Kolev V., Figliola A., Schürmann M., Başar E. (2001). Wavelet entropy: A new tool for analysis of short duration brain electrical signals. J. Neurosci. Methods.

[49] De Oliveira E.C., Tenreiro Machado J.A. (2014). A review of definitions for fractional derivatives and integral. Math. Probl. Eng..

[50] Sousa J.V.D.C., de Oliveira E.C. (2018). On the *ψ*-Hilfer fractional derivative. Commun. Nonlinear Sci. Numer. Simul..

[51] Katugampola U.N. (2016). Correction to “What is a fractional derivative?” by Ortigueira and Machado [Journal of Computational Physics, Volume 293, 15 July 2015, Pages 4–13. Special issue on Fractional PDEs]. J. Comput. Phys..

[52] Tarasov V.E. (2018). No nonlocality. No fractional derivative. Commun. Nonlinear Sci. Numer. Simul..

[53] Abdelhakim A.A., Machado J.A.T. (2019). A critical analysis of the conformable derivative. Nonlinear Dyn..

[54] Lenzi E., Mendes R., Da Silva L. (2000). Statistical mechanics based on Rényi entropy. Phys. A Stat. Mech. Appl..

[55] Parvan A., Biró T. (2005). Extensive Rényi statistics from non-extensive entropy. Phys. Lett. A.

[56] Plastino A., Casas M., Plastino A. (2000). A nonextensive maximum entropy approach to a family of nonlinear reaction–diffusion equations. Phys. A Stat. Mech. Appl..

[57] Frank T., Daffertshofer A. (2001). H-theorem for nonlinear Fokker–Planck equations related to generalized thermostatistics. Phys. A Stat. Mech. Appl..

[58] Abe S. (1997). A note on the q-deformation-theoretic aspect of the generalized entropies in nonextensive physics. Phys. Lett. A.

[59] Khinchin A.I. (1957). Mathematical Foundations of Information Theory.

[60] Shannon C.E., Weaver W. (1963). The Mathematical Theory of Communication.

[61] Lesche B. (1982). Instabilities of Rényi entropies. J. Stat. Phys..

[62] Gell-Mann M., Tsallis C. (2004). Nonextensive Entropy: Interdisciplinary Applications.

[63] Amigó J.M., Balogh S.G., Hernández S. (2018). A brief review of generalized entropies. Entropy.

[64] Namdari A., Li Z. (2019). A review of entropy measures for uncertainty quantification of stochastic processes. Adv. Mech. Eng..

[65] Abe S. (1998). Nonextensive statistical mechanics of *q*-bosons based on the *q*-deformed entropy. Phys. Lett. A.

[66] Jackson F.H. (1909). On *q*-functions and a certain difference operator. Earth Environ. Sci. Trans. R. Soc. Edinb..

[67] Akimoto M., Suzuki A. (2001). Proposition of a New Class of Entropy. J. Korean Phys. Soc..

[68] Abramowitz M., Stegun I.A. (1965). Handbook of Mathematical Functions with Formulas, Graphs, and Mathematical Tables.

[69] Ubriaco M.R. (2009). Entropies based on fractional calculus. Phys. Lett. A.

[70] Yu S., Huang T.Z., Liu X., Chen W. (2012). Information measures based on fractional calculus. Inf. Process. Lett..

[71] Radhakrishnan C., Chinnarasu R., Jambulingam S. (2014). A Fractional Entropy in Fractal Phase Space: Properties and Characterization. Int. J. Stat. Mech..

[72] Wang Q.A. (2003). Extensive generalization of statistical mechanics based on incomplete information theory. Entropy.

[73] Machado J.T. (2014). Fractional Order Generalized Information. Entropy.

[74] Bagci G.B. (2016). The third law of thermodynamics and the fractional entropies. Phys. Lett. A.

[75] Jalab H.A., Subramaniam T., Ibrahim R.W., Kahtan H., Noor N.F.M. (2019). New Texture Descriptor Based on Modified Fractional Entropy for Digital Image Splicing Forgery Detection. Entropy.

[76] Yang X.J. (2012). Advanced Local Fractional Calculus and Its Applications.

[77] Karcı A. (2016). New approach for fractional order derivatives: Fundamentals and analytic properties. Mathematics.

[78] Karcı A. (2016). Fractional order entropy: New perspectives. Optik.

[79] Ferreira R.A., Tenreiro Machado J. (2019). An Entropy Formulation Based on the Generalized Liouville Fractional Derivative. Entropy.

[80] Machado J.T., Lopes A.M. (2019). Fractional Rényi entropy. Eur. Phys. J. Plus.

[81] Beliakov G., Sola H.B., Sánchez T.C. (2016). A Practical Guide to Averaging Functions.

[82] Xu D., Erdogmuns D. (2010). Renyi’s entropy, divergence and their nonparametric estimators. Information Theoretic Learning.

[83] Van Eck N.J., Waltman L. (2010). Software survey: VOSviewer, a computer program for bibliometric mapping. Scientometrics.

